# Filling Teeth with Gold

**Published:** 1870-08

**Authors:** A. M. Moore


### FILLING TEETH WITH GOLD.

BY PROF. A. M. MOORE.

Read before the Indiana State Dental Association, June 29, 1870.

The subject of filling teeth with gold having been assigned me by the President for this occasion, needs therefore no apology for its familiarity, but however much has been written and said there is perhaps no subject in our specialty upon which greater ignorance, carelessness, and want of interest prevail; and considering the number of Dentists, there never was a time in the history of the Dental profession when so few first class operations were performed.

The question naturally arises? "Why is this?" Are we slumbering in this age of progress?" I answer no.

For the last quarter of a century the subject of filling teeth with gold has been gradually growing in interest to the scientific and progressive Dentist; and especially within the last few years such gigantic strides have been made in this branch of Dental surgery that the Dentist whose operation does not vie with nature itself, must needs retire ingloriously from the professional field to one more in keeping with his limited abilities, or endure the oprobrium of a "bungler;" or the more loathsome epithet, "an excrescence hanging somewhere on the verge of the profession." But it is not in the province of this paper to divide the dental profession, or operators, into different classes, for it is very obvious that all can not attain alike the same degree of perfection. Yet what seems to be wanting, in many, is that *interest* in the welfare of their patient that would lead them into a higher field of operation.

When I say vie with nature, I mean to be understood that when the operation of filling a tooth is completed it will be throughout, in such perfect harmony and relationship with bone itself that we have, in a measure, the original tooth, with its strength, form, and beauty. Not a fissure, not a prominence wanting! All the curves and angles that the great Creator formed and carved out in His infinite wisdom, still preserved. Then it is that we have nature and art so completely blended that it can not be told where nature leaves off or where art commences.

Let us take for an example the right inferior first molar. the decay in the anterior approximal surface extending to the anterior crown pit, running each way transversely through buccal fissures to buccal pit, and lingual fissure and posterior buccal fissure, and posterior fissure. The first step should be a thorough examination with a concave mirror and sharp pointed instrument, along the entire course of the decay.

By means of a drill and gouge the central cavity should be enlarged, and with enamel chisel the fissures running out of the same, with all sharp angular points removed, making the way for the file, which is the most convenient and useful instrument when it can be used in a file carrier; first to the fissures extending to the buccal surface, and the thin wall intervening to buccal pit wholly cut away; making a free communication with central cavity, and the same process with opposite or lingual fissures.

The file not being applicable to the fissures extending from central to approximal surface, on account of adjacent teeth, the enamel chisel and great curve fissure instrument are best adapted, and most convenient.

All fissures and cavities, when prepared, have perfectly parallel walls and communicating with each other, making one continuous cavity. Having the cavity all properly prepared, the next step is the adjustment of the rubber dam. And now allow me to remark, that all operators use the rubber dam, for it is all that is claimed for it, and can not be too highly recommended. Let Dr. Barnum have all the credit for bringing it to the notice of the Dental profession. To adjust the dam the chair should be thrown well back, and the operator at the back of the chair; it is first put on the second bicuspid, the first molar and last by the second molar. The ligatures of floss silk should be about twelve inches in length, and well waxed, and drawn around each one of the teeth above mentioned. Some Dentists prefer to tie the cord. This, in my judgment, is not best as a rule. If it properly catches the rubber, and with a little see-saw motion of the cord it is buried between gum and tooth, and made absolutely water-tight, no more can be gained if the silk is tied, and a fair chance to loose. The rubber being adjusted it may be held out of the way of the operator by means of an appliance for the purpose. With instruments all ready to proceed it may be well to give them a passing notice. Several foot instruments of different sizes, with from four to six rows of serration, very fine, should be used. The best instruments that are in the market, I believe, were first recommended and used by Dr. Varny, of New York, whose unsurpassed operations have been accepted as the beau ideal, which all should aspire to equal.

Some of his finest instruments have four rows of minute serrations, while others are armed with twenty. I understand that Dr. Varny has been using these fine serrated plugger for a number of years; and to this, in a large measure, may be attributed the wonderful approximation to perfection in his fillings. After carefully drying the cavity the next step is the introduction of the gold, which may be Nos. 4 or 6, rolled up from a whole or half sheet, and cut into pellets. The first pellet is carried with foil pliers to the bottom of the posterior buccal fissure, thoroughly condensed with lead or steel mallet; other pellets are added and the entire fissure filled up to central cavity, the same with the posterior fissu running over the balance of central cavity into transverse fissure each way, to their extremities.

Having proceeded thus far the anterior approximal surface should be commenced, from some retaining point, and built up squarely, connecting with the central, which is the place to finish. Much merit has been claimed by some of our most popular operators for heavy foil; especially in the latter part of the operation, and in building up sharp angles, where great strength and density is required. There is a degree of plausibility in it, and it will have the best experience. The heavy foils weld more readily, and appear to be denser than a plug from the lighter foils. No more skill is required for working them than the ordinary numbers, only that the Dentist should begin his experience with No. 10, and gradually work up to 120; which is probably about as high as is expedient to go. No. 120 should be cut into square plats, single thickness, a little larger than the cavity, and laid on flat, condensed over the surface, allowing that which overlaps to fold down around the border.

A higher degree of finish is attained with the thick foils, and a surface that is more durable, and consequently should be used, as uniformly as possible. A better quality of instruments are required to work it; which may be the means of stimulating many of our Dentists to use finer and better instruments.

